# Reversible Posterior Encephalopathy Syndrome Secondary to Sunitinib

**DOI:** 10.1155/2014/952624

**Published:** 2014-05-13

**Authors:** Ricardo Costa, Rubens Costa, Renata Costa, Gilberto Moura de Brito Junior, Henrique Queiroz Cartaxo, Alex Caetano de Barros

**Affiliations:** ^1^Department of Oncology, Real Hospital Portugues, 52010 Recife, PE, Brazil; ^2^Cedar Valley Cancer Center, Waterloo, IA 50701, USA; ^3^American University of the Caribbean, Coral Gables, FL 33134, USA; ^4^Department of Radiology, Real Hospital Portugues, 52010 Recife, PE, Brazil; ^5^Department of Neurosurgery, Real Hospital Portugues, 52010 Recife, PE, Brazil

## Abstract

Reversible posterior leukoencephalopathy syndrome (RPLS) is clinical radiologic condition associated with neurological symptoms and cerebral white matter edema. It has been associated with uncontrolled hypertension, eclampsia, immunosuppressants, and more recently the use of antiangiogenic drugs. Sunitinib is an inhibitor of the vascular endothelial growth factor receptor widely used in the treatment of metastatic renal cell carcinoma (RCC). We report a rare case of RPLS occurring on therapy with sunitinib in a patient with RCC. Our aim is to highlight the importance of considering RPLS as a diagnostic possibility and to hold sunitinib for RCC patients presenting with neurologic symptoms.

## 1. Introduction

Reversible posterior leukoencephalopathy syndrome (RPLS) is clinical radiologic condition associated with neurological symptoms and white matter edema. RPLS is characterized by cerebral autoregulation and endothelial dysfunction secondary to a host of etiologies [1]. RPLS has been associated with uncontrolled hypertension, eclampsia, immunosuppressive drugs [2–4], and more recently antiangiogenic drugs [5–7].

An estimated 65,150 Americans were diagnosed with renal cell carcinoma and 13,680 died of the disease in the United States in 2013. Renal cell carcinoma comprises about 4% of all cases of cancer [8]. The five-year survival rate for distant metastatic disease is approximately 12.3% based on analysis of the SEER data from 2003 to 2009 [9].

Sunitinib is a tyrosine kinase inhibitor which inhibits vascular endothelial growth factor receptor. It has been widely used in the upfront treatment of metastatic renal cell carcinoma improving patient outcome [10]. We report a case of RPLS occurring on therapy with sunitinib in patient with metastatic renal cell carcinoma (RCC).

## 2. Case

The patient was a 67-year-old male who presented with dry persistent cough for approximately one month. He was hospitalized for worsening dyspnea, cough, and clinical deterioration. Computerized tomography (CT) scan of the chest and abdomen showed a large right pleural effusion, a right renal mass, and multiple lung and liver nodules. The patient underwent a CT-guided biopsy of one of the lung nodules to establish a definite diagnosis. Pathological and immunohistochemical analyses were consistent with renal cell carcinoma of clear cell histology.

He was treated with sunitinib 50 mg orally daily for 4 weeks on a 6-week cycle. He experienced a rapid clinical response with improvement of respiratory symptoms. A restaging CT scan of the chest after cycle 1 of treatment showed evidence of necrosis of multiple lung nodules indicating at least stable disease.

He presented to the emergency department with complaints of headache and amaurosis 19 days after starting cycle 2 of treatment on previous schedule and dosing. His blood pressure was elevated at 180/100 mmHg despite no previous documented hypertension to that moment. On physical exam, he was found to be disoriented. Lateral nystagmus and visual field defect were appreciated. The remainder of the neurologic examination was normal.

**Figure 1:**
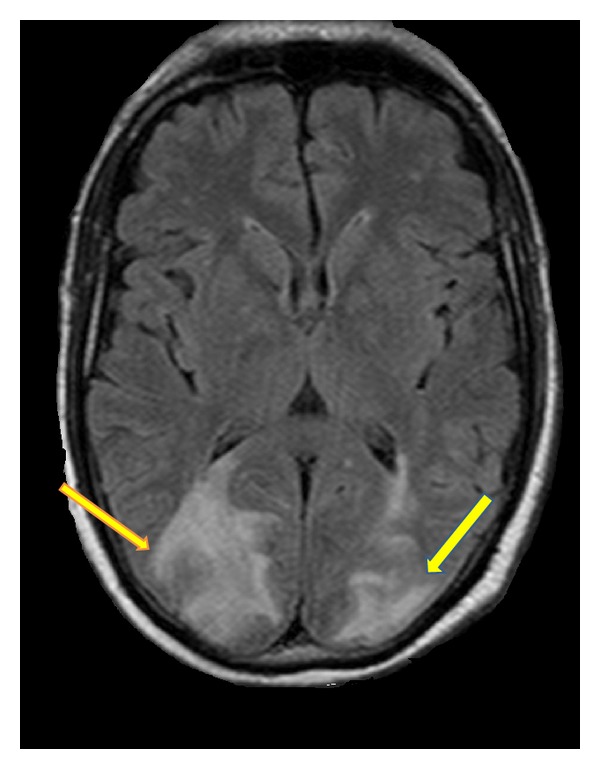
Increased T2 uptake sequence on cerebral occipital parietal regions.

**Figure 2:**
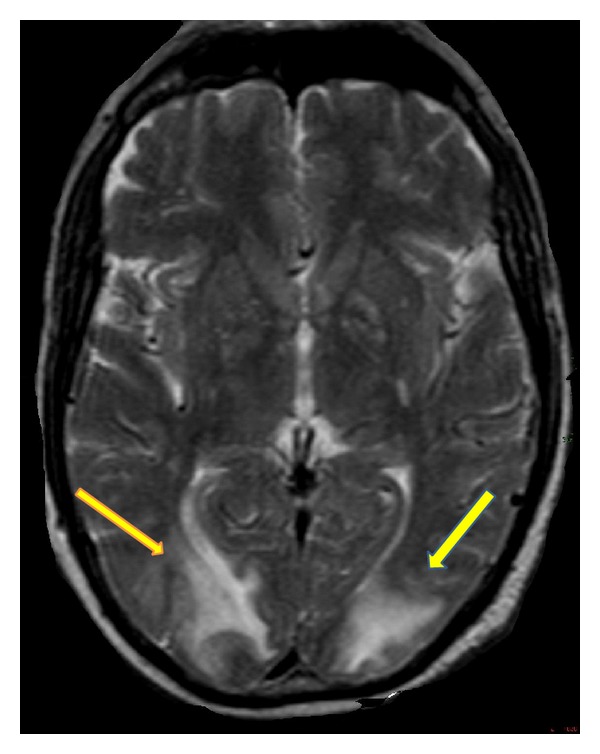
Increased fluid-attenuated inversion recovery (FLAIR) uptake sequence on cerebral occipital parietal regions.

Magnetic resonance imaging (MRI) of the central nervous system showed edema like increased T2 and fluid-attenuated inversion recovery (FLAIR) sequence uptake on parietal occipital white matter bilaterally (Figures 1 and 2).

Sunitinib was stopped. Intravenous benzodiazepine and anticonvulsants were started. Antihypertensives were given in an attempt to control blood pressure levels.

The patient gradually improved over the course of several weeks after discontinuation of sunitinib. The medication was not restarted given concerns of recurring side effects. After resolution of symptoms, second-line therapy with sorafenib was initiated with no clinical benefit.

## 3. Discussion

Sunitinib is a tyrosine kinase inhibitor which showed improved outcomes in RCC including progression-free survival and response rates when compared to previous standard biologic therapy interferon [10].

RPLS is a rare condition which has been associated with new cancer antiangiogenic therapy. Its pathophysiology and true incidence remain unknown in this setting. If recognized and treated in a timely fashion, the symptoms and radiologic abnormalities are almost always reversible. When unrecognized, it may progress to ischemia, massive infarction leading to death [18].

Reports of sunitinib induced RPLS are scarce. Its onset may vary from several days to months after start of therapy. Fortunately, symptoms resolve in a matter of days to several weeks when managed appropriately (Table 1) [11–17].

In summary, RPLS is most often reversible with prompt management. However, it should be treated as a life-threatening event, which medical oncologists should be aware of, look out for, and promptly treat once diagnosis is established or suspected.

## Figures and Tables

**Table 1:** Reports of reverse posterior leukoencephalopathy syndrome associated with sunitinib.

Case author	Age (years)/gender	Onset after starting sunitinib	Management	Recovery after discontinuation
Padhy et al. [11]	65/male	8 days	Sunitinib discontinuation, antihypertensive	Complete recovery in 17 days

Kapiteijn et al. [12]	54/female	8 months	Sunitinib discontinuation, antihypertensive, anticonvulsants	Complete recovery in 10 days

Martín et al. [13]	70/female	2 weeks	Sunitinib discontinuation, antihypertensive, anticonvulsants	Complete recovery in few days

Cumurciuc et al. [14]	39/female	1 week	Sunitinib discontinuation, antihypertensive, anticonvulsants	Complete recovery in 2 weeks

Chen and Agarwal [15]	48/female	1 week	Sunitinib discontinuation	Complete recovery in 3 weeks

van der veldt et al. [16]	84/female	14 days	sunitinib discontinuation	Complete recovery in 3 days

van der veldt et al. [16]	74/male	13 days	sunitinib discontinuation	Complete recovery in 3 days

Hadj et al. [17]	61/male	15 weeks	Sunitinib discontinuation, antihypertensive, anticonvulsants	Complete recovery in 10 weeks

Present case	67/male	2 months	Sunitinib discontinuation, antihypertensive, anticonvulsants	Complete recovery not achieved (patient deceased few weeks after discontinuation of sunitinib due to cancer progression)
